# Patient satisfaction after total knee arthroplasty is better in patients with pre-operative complete joint space collapse

**DOI:** 10.1007/s00264-018-4185-3

**Published:** 2018-10-02

**Authors:** Michael Liebensteiner, Alexander Wurm, Dennis Gamper, Wilhelm Oberaigner, Dietmar Dammerer, Martin Krismer

**Affiliations:** 1 Department of Orthopaedic Sugery, Medical University of Innsbruck, Anichstrase 35, 6020 Innsbruck, Austria; 2 Medical University of Innsbruck, Innsbruck, Austria; 3 Department of Clinical Epidemiology, Tirol Kliniken, Innsbruck, Austria; 4 Department of Public Health, Health Services Research and Health Technology Assessment, Institute of Public Health, Medical Decision Making and HTA, UMIT the Health & Life Sciences University, Hall i.T., Austria

**Keywords:** Total knee arthroplasty, Joint space narrowing, Joint space width, Osteoarthritis, Total knee replacement, Outcome

## Abstract

**Aim of the study:**

To determine if pre-operative radiologic minimal joint space width (mJSW) is related to the outcome of total knee arthroplasty (TKA) (primary hypothesis). Likewise, the aim was to test if pre-operative mJSW is related to prosthesis survival (secondary hypothesis).

**Methods:**

A retrospective comparative analysis was performed. Group 1 was comprised of patients with pre-operative mJSW 0–1 mm. Group 2 were patients with pre-operative mJSW ≥ 2 mm. The clinical outcome was determined with the Western Ontario and MacMaster Universities Osteoarthritis Index (WOMAC) score pre-operatively and one year after TKA. Only patients with pre-operative weight-bearing radiographs and complete WOMAC score data were accepted.

**Results:**

Available for analysis were 377 patients, of whom 188 were allocated to Group 1 (118 female, 70 male, age 70 ± 11 years) and 189 to Group 2 (118 female, 71 male, age 70 ± 13 years). Pre-operative WOMAC total and WOMAC subscores showed no significant differences between groups. Post-operatively, the WOMAC total was significantly better in Group 1 than in Group 2, 10 ± 22 and 19 ± 31, respectively (*p* < 0.001, Power 97.5%). Similarly, the WOMAC subscores for pain, stiffness, and function were also significantly better in Group 1 than in Group 2. Five-year prosthesis survival was 94.2 and 91.6% in Groups 1 and 2, respectively (*p* = 0.07, Power 71%).

**Discussion:**

Patients with pre-operative complete joint space collapse (0 to 1 mm mJSW) achieve a significantly better WOMAC result from TKA than do those with a mJSW equal to or greater than 2 mm. From our findings, it is recommended that “complete joint space collapse” especially be used as an indication for TKA surgery.

**Conclusion:**

Our study was underpowered to sufficiently show an effect of pre-operative mJSW on prosthesis survival.

**Electronic supplementary material:**

The online version of this article (10.1007/s00264-018-4185-3) contains supplementary material, which is available to authorized users.

## Introduction

Up to 30% of patients were reported to not be satisfied with the outcome of total knee arthroplasty because of unexplained pain (TKA) [1–5]. When discussing patient dissatisfaction following TKA, a differentiation can be made between surgery-related [6], implant-related and patient-related factors [7]. Among other patient-related factors, the severity of knee osteoarthritis (OA) (e.g. joint space width) is of obvious importance.

Merle-Vincent et al. investigated the influence of pre-operative severity of knee OA on patient satisfaction after TKA in 264 cases [8]. They reported that patients with pre-operative more severe joint space narrowing were more likely to be satisfied two years post-operative. Five other studies also investigated the relationship between severity of knee OA and TKA outcome [5, 9–12]. These studies analyzed the pre-operative radiographic OA severity in terms of Kellgren-Lawrence Gradings (KL Grade). The findings of those studies were highly conflicting. While three found better TKA outcome (knee scores, pain, quality of life) in patients with more severe OA [5, 9, 11] the others found no such associations [10, 12]. However, the measurement of radiologic OA severity applied in the latter studies (KL Grade) was reported to be inferior to the measurement of joint space width regarding reliability and validity [13, 14]. In addition, many of the above-mentioned studies applied less robust outcome measurements (e.g. patient satisfaction, pain).

In summary, there is no consensus on the influence of pre-operative radiographic severity of knee OA on the outcome of TKA [10].

It was the aim of the study to investigate whether patients with different OA severity also differ with regard to outcome following TKA (knee score and prosthesis survival). In light of the shortcomings of previous studies our study approach aimed to incorporate the following: a) high case numbers from data extraction from the Tyrolean State Arthroplasty Registry, b) a reliable assessment method of OA severity (joint space width, JSW) and c) robust clinical outcome measurements (WOMAC score and prosthesis survival).

It was hypothesized that patients without complete radiologic joint space collapse would experience a different clinical knee score outcome (WOMAC score) than would those with complete radiologic joint space collapse (primary hypothesis). It was also hypothesized that these two samples would differ with regard to prosthesis survival (secondary hypothesis).

## Materials and methods

A retrospective comparative design was applied. Data already available from clinical routine were analyzed after approval by the Ethics Committee of the Medical University (approval no. AN2017-0021-370/4.1). Patients who previously underwent primary TKA as part of the clinical routine were analyzed. Cases were excluded in the case of the following: a) incomplete WOMAC data, b) primary prostheses other than cruciate-retaining and c) missing pre-operative Schuss-view radiograph.

Cruciate-retaining TKA was performed in all cases (232 Scorpio-CR and 145 Triathlon-CR, both Stryker, Kalamazoo, MI, USA). The prosthesis was implanted according to the manufacturer’s instructions using a measured resection technique and standard cutting blocks and instruments. Intramedullar referencing was applied at the femur and extramedullar referencing at the tibia. In accordance with the clinical routine at our institution, the patella was left unresurfaced. All operations were performed by consultant orthopaedic surgeons specialized in knee arthroplasty or under the supervision of one of these surgeons. Patient positioning, antibiotic and deep vein thrombosis prophylaxis, draping and tourniquet control were standardized. All patients underwent the same standardized rehabilitation program after surgery. Patients were mobilized from the first post-operative day under supervision of our physiotherapists. Exercises included continuous passive motion, assisted and unassisted knee extension, walking and stair climbing with two crutches and progression as tolerated.

**Fig. 1:**
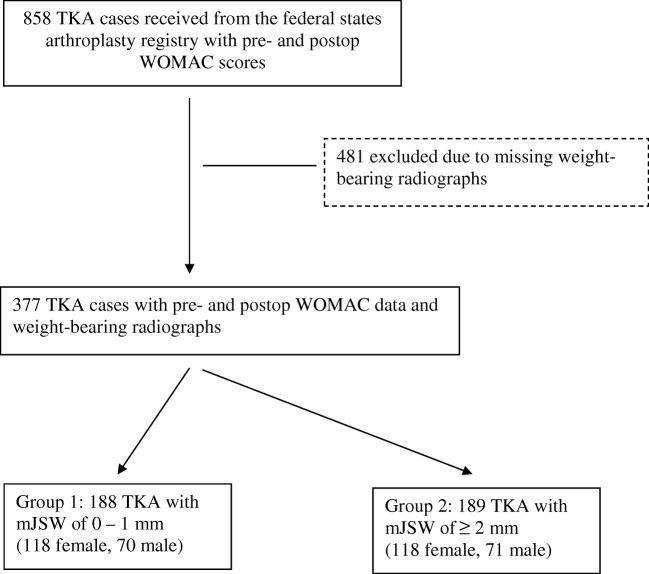
Flow of patients considered for enrollment (*TKA* total knee arthroplasty, *mJSW* minimal joint space width, *WOMAC* Western Ontario and MacMaster Universities Osteoarthritis Index)

**Fig. 2:**
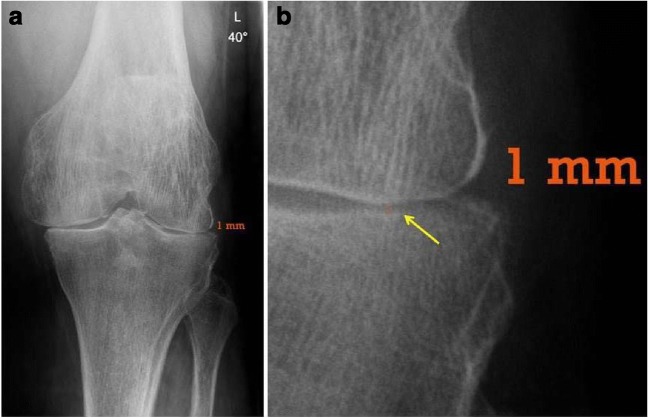
Example of weight-bearing flexed radiographs (Schuss-view) and of the measurement of the most pronounced narrowing of the joint space width; **a** overview; **b** detail

Joint space width was determined from radiographs from the university hospital’s PACS by always the same investigator using the same software (Impax EE, Agfa Health Care N.V., Mortsel, Belgium). Amongst different means of radiographically determining severity of knee OA, previous studies recommended the measurement of joint space width due to superior reliability and validity as compared to other methods [13, 14]. From weight-bearing flexed radiographs (Schuss-view) [15–17], the location of the most pronounced narrowing of the joint space width was identified (Figs. 1 and 2). The joint space was measured to one decimal of a millimeter at that point to determine the parameter “minimal joint space width (mJSW)”. In the case of not just full joint space collapse but even bony defects (e.g. femoral condyle eroding in the tibia), mJSW was defined as 0 mm because measurement of negative values would have been less accurate. The measured values of mJSW were rounded to full millimeters and patients were assigned to Group 1 if mJSW was 0 or 1 mm, and to Group 2 if mJSW was ≥2 mm.

For patient-reported outcome measurement, the Western Ontario and MacMaster Universities Osteoarthritis Index (WOMAC) score [18] was available as part of quality control issues. It was applied in the German-language version [19] (main outcome parameter). The questionnaire was completed the day before surgery and again post-operatively one year after surgery. The WOMAC questionnaire collects data on pain, stiffness and physical function. Every item was completed on an 11-point scale and converted for analysis purposes to a scale from 0 to 100%, 0 denoting the best and 100% the worst response. The score for each of the three main dimensions is defined as the sum of all item scores divided by the number of items. The total score was defined as the sum of pain, stiffness and function scores divided by three.

Available were 858 cases with full WOMAC data. However, 481 had either no pre-operative weight-bearing X-ray at all or only a weight-bearing whole leg radiograph. That left 377 patients with full data sets available for analysis. Group 1 contained 188 patients (118 female, 70 male, 70 ± 11 years, BMI 29.5 ± 5.6). Group 2 consisted of 189 patients (118 female, 71 male, age 70 ± 13 years, BMI 29.8 ± 5.1).

Data analysis was performed with SPSS Version 24 (International Business Machines Corporation, Armonk, NY, USA) and with Stata Version 13 (StataCorp LP, 4905 Lakeway Drive, College Station, TX 77845, USA) for Kaplan-Meier analysis, see below. Data was not normally distributed, as indicated by the Kolmogorov-Smirnov test. As descriptive values medians and interquartile ranges were determined. The Mann–Whitney U tests were applied to test for significant differences between groups regarding the WOMAC total score and the WOMAC subscores. Alpha was defined as 0.05 (two-tailed). A post-hoc power analysis revealed a power of 98% for the WOMAC total score.

With regard to our secondary hypothesis, we estimated cumulative revision-free survival from date of surgery until date of revision, date of death or end of follow-up (31 Dec 2015), whichever occurred first, by applying the Kaplan-Meier method. Differences in survival curves were tested using the generalized Fleming-Harrington test of equality, with parameters *q* and *p* chosen at *p* = 0.0, *q* = 0.03. A post-hoc power analysis for the secondary hypothesis revealed a power of 71%.

## Results

Pre-operative WOMAC total and WOMAC subscores showed no significant differences between Group 1 and Group 2 (Table 1). Post-operatively, the WOMAC total was significantly better in Group 1 than in Group 2, 10 ± 22 and 19 ± 31, respectively (*p* < 0.001, primary hypothesis). Similarly, the WOMAC subscores pain, stiffness and function were also significantly better in Group 1 than in Group 2 (see Table 1 for details).

**Table 1:** Pre- and post-operative values for WOMAC pain, WOMAC stiffness, WOMAC function and WOMAC total

	Group 1 (mJSW: 0–1 mm)		Group 2 (mJSW: ≥ 2 mm)		
	Md	IQR	Md	IQR	*p* value
WOMAC pain preop [%]	50	28	50	31	0.551
WOMAC stiffness preop [%]	55	35	55	40	0.982
WOMAC function preop [%]	49.5	32	52	28	0.298
WOMAC total preop [%]	52	28	53	29	0.539
WOMAC pain 1 y [%]	6	16	14	27	< 0.001
WOMAC stiffness 1 y [%]	15	25	25	38	0.003
WOMAC function 1 y [%]	9.5	22	20	33	< 0.001
WOMAC total 1 y [%]	10	22	19	31	< 0.001

*Md* median, *IQR* inter-quartile-rangem, *JSW* minimal joint space width, *y* year, *preop* preoperative

**Fig. 3:**
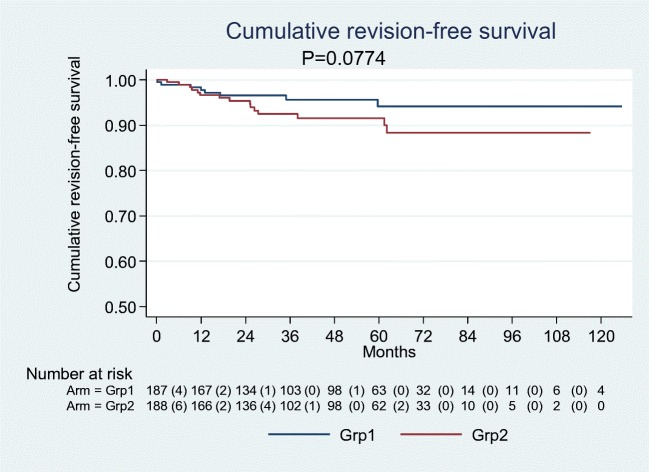
Prosthesis survival in the two groups. Group 1: pre-operative joint space width of 0–1 mm, Group 2: pre-operative joint space width ≥ 2 mm. *Grp* group

Regarding the secondary hypothesis, five year prosthesis survival was 94.2% and 91.6% in Groups 1 and 2, respectively (*p* = 0.07) (Table 3, Fig. 3). Mean follow-up time was 3.8 years.

## Discussion

With regard to our hypothesis, the most important finding was that patients with virtually an absolute joint space collapse (0 to 1 mm mJSW) achieved a significantly better result than did those with a mJSW equal to or greater than 2 mm. Clearly, our hypothesis was confirmed. Group 1 had a post-operative WOMAC total score that was 9% better than that of Group 2 (absolute difference, *p* < 0.001). This difference was greater than what previous investigators determined as a minimal clinically important difference for the WOMAC score [20]. The same is true for the differences between the groups in all the WOMAC subscores.

Trying to compare our findings with what previous researchers found (see Table 2 for an overview), it appears that part of our findings are supported by those of Valdes et al. [11]. They reported that low preoperative radiologic OA severity led to more post-operative pain as determined with the WOMAC pain score. However, the measurement of radiologic OA severity applied in that study (Kellgren-Lawrence Grade) was reported to be inferior to the measurement of mJSW regarding reliability and validity [13, 14]. Our findings are also in good agreement with those of Merle-Vincent et al. [8]. They reported that patients with pre-operative severe joint space narrowing were more likely to be satisfied two years post-operative. It is regarded as a strength of the study by Merle-Vincent that they *prospectively* collected data on 264 patients. However, their method of defining values greater than 50 on a satisfaction scale of 0–100 as‚ “good satisfaction” might invite criticism. The WOMAC score as used in our study is regarded as a better outcome measuring tool. Also, Keurentjes et al. analysed the influence of pre-operative radiographic severity (Kelgren-Lawrence-Scale) of knee OA on patient satisfaction and quality of life [5]. Both satisfaction and quality of life were reported to be better in those with more severe OA, which again supports the findings made in our study. Similar findings were provided by Polkowski et al., who reported that their TKA patients showed a pre-operative more severe stage of knee OA (Kellgren-Lawrence Grade) that was associated with less post-operative knee pain [9]. Conflicting results were reported by Tilbury et al. [10]. In their prospective study, no associations were determined between pre-operative radiographic severity of knee OA (Kellgren-Lawrence Grade) and one year post-operative knee outcome. It might be speculated whether they failed to identify an effect of pre-operative radiographic severity on the outcome because a less favourable radiographic knee OA scale was applied [13, 14]. Also Vina et al. reported results that contrast with ours [12]. Patients who improved above the CMID threshold (clinically minimal important difference) were compared with those who did not. According to the authors, pre-operative radiographic OA severity did not differ between these two groups. Trying to take together the contributions of other researchers and the current study, the results seem to be in favour of the existence of an association between pre-operative OA grade and TKA outcome (5 studies pro: 2 studies against; see Table 2).

**Table 2:** Overview of previous studies also investigating for an association between pre-operative OA severity and outcome of total knee arthroplasty

Author	Ref.	N	Radiographic method of preop OA assessment	Outcome parameters	Findings
Vina	[21]	269	K-L grade	WOMAC total	No association
Keurentjes	[11]	278	K-L grade	Patient satisfaction health-Related quality of life (SF-36)	More severe OA: better outcome
Valdes	[22]	860	K-L grade	WOMAC pain	More severe OA: better outcome
Tilbury	[23]	271	K-L grade	KOOS, OKS	No association
Merle-Vincent	[15]	264	minimal JSW in weight-bearing x-rays	Patient satisfaction	More severe OA: better outcome
Polkowski	[17]	309	K-L grade	Pain	More severe OA: better outcome
This study	n/a	377	minimal JSW in weight-bearing x-rays	WOMAC	More severe OA: better outcome

*n* number of cases, *K-L grade* Kellgren–Lawrence grade, *WOMAC* Western Ontario and MacMaster Universities OA Index, *OA* osteoarthritis, *OKS* Oxford Knee Score, *KOOS* Knee Injury and Osteoarthritis Outcome Score, *preop* preoperative

Regarding the secondary hypothesis, five year prosthesis survival was 94.2 and 91.6% in Groups 1 and 2, respectively (*p* = 0.07) (Table 3, Fig. 1). With a power value of 71%, these findings are regarded as underpowered. Future studies might include more patients when re-evaluating that issue. We intentionally did not perform an a priori sample size calculation. Instead, we included *all* available patients in the retrospective analysis and performed the power calculation post-hoc. Unfortunately, none of the above-mentioned studies analyzed whether prosthesis survival was related to the pre-operative OA severity grade.

**Table 3:** Prosthesis survival in the two groups. Group 1: pre-operative joint space width of 0–1 mm, Group 2: preoperative joint space width ≥ 2 mm

	FU time	Survival	[95% CI]
Group 1			
1 y	0.9779	0.9421	0.9917
2 y	0.9656	0.9249	0.9844
3 y	0.9563	0.9091	0.9793
4 y	0.9563	0.9091	0.9793
5 y	0.9416	0.8797	0.9722
Group 2			
1 y	0.9664	0.9267	0.9848
2 y	0.9539	0.9098	0.9767
3 y	0.9251	0.8712	0.9570
4 y	0.9160	0.8585	0.9508
5 y	0.9160	0.8585	0.9508

*CI* confidence interval, *FU* follow-up

The findings made in our study also raise the question how to handle those requesting TKA despite a still relatively large mJSW. We recommend considering the following three aspects: first, an MRI should be performed to rule out other pathologies that may be missed with only X-rays (e.g. symptomatic meniscus tear in someone with mild OA). Second, in patients with mild radiographic OA, additional factors (e.g. depression) may boost the pain from knee OA and should be addressed [23].

The following limitations of the study are acknowledged. First, it was a retrospective study with the typical weaknesses associated with such studies: selection bias, information bias, inability to investigate parameters other than those previously collected during clinical routine, reliance on data collected by others etc. Second, although previously suggested [22], we did not succeed in collecting physical activity data and health-related quality of life data in conjunction with the knee-specific WOMAC data. Third, as mentioned above, our study must be regarded as underpowered with respect to our secondary hypothesis. However, no further patients would have been available with both complete WOMAC and X-ray data (*n* = 377). So an a-priori sample size calculation would not have solved that problem.

It is regarded as a strength of our study that radiographic severity of knee OA was—in contrast to most previous studies—assessed in terms of mJSW, which was found to be the preferable method [13, 14]. Other strengths are the use of an established outcome parameter (WOMAC) and the second highest case number of available studies (see Table 2).

The study findings are regarded as of high clinical relevance. Particularly high patient satisfaction can be expected when using “complete joint space collapse” as indication for TKA surgery. In the case of incomplete joint space collapse, further conservative therapy [21, 24] or joint preserving knee surgery [25] might be considered an alternative to TKA.

## Conclusions

Patients with pre-operative complete joint space collapse (0 to 1 mm mJSW) achieve a significantly better WOMAC result from TKA than do those with a mJSW equal to or greater than 2 mm. On the basis of our findings, it is recommended that “complete joint space collapse” especially be used as indication for TKA surgery. Our study was underpowered to sufficiently show an effect of pre-operative mJSW on prosthesis survival.

## Electronic supplementary material

ESM 1 (DOCX 57 kb)

ESM 2 (DOCX 59 kb)
