# Supplementary material for: Patient satisfaction after total knee arthroplasty is better in patients with pre-operative complete joint space collapse
Source: Int Orthop. 2018 Oct 2;43(8):1841–7. doi: 10.1007/s00264-018-4185-3 (PMC6647264; doi:10.1007/s00264-018-4185-3)
Supplement: Supplementary file 2 — (DOCX 59 kb) [file 264_2018_4185_MOESM2_ESM.docx]

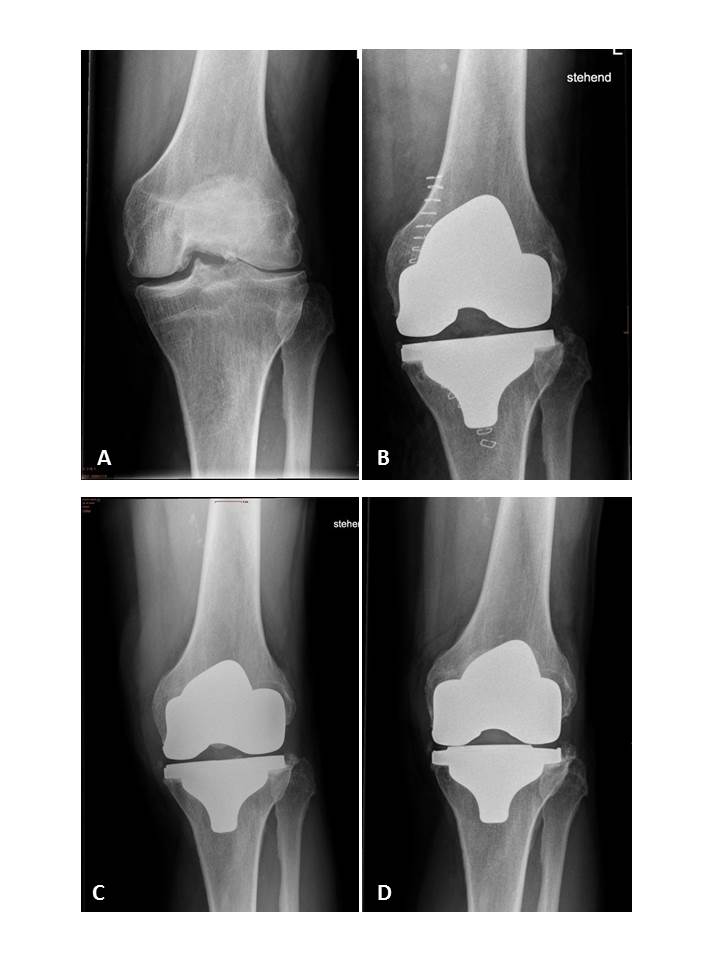
**Supplementary Material 2.** Pre-operative (A) and post-operative X-rays after one month (B), five months (C) and six months (D). Example patient Group 2
